# Supplementary material for: MetaRibo-Seq measures translation in microbiomes
Source: Nat Commun. 2020 Jun 29;11:3268. doi: 10.1038/s41467-020-17081-z (PMC7324362; doi:10.1038/s41467-020-17081-z)
Supplement: Supplementary file 10 — Supplementary Data 7 [file 41467_2020_17081_MOESM10_ESM.zip › File2/Confidence_High_Taxonomy/230872_out.krona.html]

Javascript must be enabled to view this page.

members
magnitude
magnitudeUnassigned
count
unassigned
taxon
rank

230872\_out

2

superkingdom
2
2

2
1239
phylum

class
186801
2

2
order
186802

1

SRS052697\_contig\_number\_43060
1897043
species

541000
family
1

1
species
1898205

SRS142503\_contig\_number\_44986
